# Advances in Sources, Content Determination, and Bioactivity of 20α-Hydroxyprogesterone

**DOI:** 10.2174/0118715303302957250117052100

**Published:** 2025-02-10

**Authors:** Liangyun Li, Shujing Yan, Yuexuan Cheng, Chunhong Zhong, Chunli Chen, Xiaoli Gao

**Affiliations:** 1 College of Pharmacy, Xinjiang Medical University, Urumqi, Xinjiang, 830017, China;; 2 Engineering Research Center of Xinjiang and Central Asian Medicine Resources, Ministry of Education, Urumqi, Xinjiang, 830017, China;; 3 Xinjiang Key Laboratory of Active Components and Drug Release Technology of Natural Drugs, Urumqi, Xinjiang, 830017, China;; 4 Xinjiang Key Laboratory of Biopharmaceuticals and Medical Devices, Urumqi, Xinjiang, 830017, China

**Keywords:** 20α-hydroxyprogesterone, progesterone, endogenous sources, exogenous sources, content determination method, biological activity, pharmacokinetic characteristics

## Abstract

20α-hydroxyprogesterone [(20*S*)-20-hydroxypregn-4-en-3-one, 20α-DHP] is one of the endogenous metabolites of progesterone (Pregn-4-ene-3,20-dione, P4) and a steroid hormone. The literature related to 20α-DHP mainly concentrates on the years from the 1950s to 1970s, and a review of 20α-DHP has not been conducted. In this work, the endogenous and exogenous sources of 20α-DHP are introduced, and methods for determining 20α-DHP in biological samples are described. The biological activities of 20α-DHP are summarized in detail, including the maintenance of pregnancy, endometrial protection, regulation of hormone secretion, ovulation promotion, uterine epithelial cell proliferation, antagonism of breast cancer, and as a diagnostic indicator for psoriasis and polycystic ovarian syndrome. Finally, the pharmacokinetic characteristics of 20α-DHP are briefly introduced to provide a reference for the further development and utilization of 20α-DHP.

## 1 INTRODUCTION

Progestins are steroidal compounds with progestational activity, which are widely used in treating female gynecological and endocrine disorders, such as menopausal hormone therapy and contraception [1, 2]. Currently, progestins are mainly classified into two major categories, natural and synthetic, of which progesterone (Pregn-4-ene-3,20-dione, C_21_H_30_O_2_, P4) is the only natural progestin, which is mainly secreted by the corpus luteum of the ovary and is necessary for the maintenance of pregnancy [3, 4]. In addition, it is also widely used to treat diseases, such as menstrual disorders, premenstrual syndrome, luteal insufficiency, threatened abortion, and habitual abortion [5, 6]. In early studies to explore the P4 metabolic pathway, Zander *et al.* [7] discovered 20α-hydroxyprogesterone [(20*S*)-20-hydroxypregn-4-en-3-one, 20α-DHP] in 1954, which was followed by the discovery of another metabolite besides 20α-DHP, 20β-hydroxyprogesterone [(20*R*)-20-hydroxypregn-4-en-3-one, 20β-DHP] in 1959 [8]. The physical properties of 20α-DHP and 20β-DHP are as follows: molecular weight: 316.47800, density: 1.09 g/cm^3^, boiling point: 451.7°C at 760 mmHg, Log P: 4.51530, white or crystalline powder, odorless, tasteless; dissolved in ethanol, ether or vegetable oil, and insoluble in water. The main differences between the ^1^H-NMR data of the two diastereoisomers are as follows [9, 10]: ^1^H NMR (200.13 MHz): 20α-DHP: δ 0.70 (3H, s) (Me-18), 1.18 (3H, s) (Me-19), 1.23 (3H, d, *J*=6.3 Hz) (Me-21), 3.72 (1H, m) (H-20), 5.72 (1H, br d) (H-4); 20β-DHP: δ 0.78 (3H, s) (Me-18), 1.14 (3H, d, *J*=6.3 Hz) (Me-21), 1.23 (3H, s) (Me-19), 3.72 (1H, m) (H-20), 5.75 (1H, br d) (H-4).

The two enzymes that metabolize P4 are 20α-hydroxysteroid dehydrogenase (20α-HSD) and 20β-hydroxysteroid dehydrogenase (20β-HSD), which metabolize P4 to 20α-DHP and 20β-DHP, respectively [11-13]. During the 1950s and 1960s, researchers evaluated progestational activity mainly through Hooker-Forbes and Clauberg tests based on the principle that P4 can produce specific effects on the uterine horn [8]. In 1964, Hilliard *et al.* [14] demonstrated that the surge effect of 20α-DHP was related to the release of gonadotropins from the pituitary gland *in vivo*, indicating 20α-DHP to be involved in hormone regulation. In 2002, Nowak *et al.* [15] found that 20α-DHP could selectively enter or remain in the central nervous system after injection through the tail vein, which means that 20α-DHP may have physiological effects on the central nervous system.

Studies on the activity of 20α-DHP have mainly focused on the 20^th^ century, and in the 21^st^ century, there have been few related research reports; with respect to 20β-DHP, from the discovery to the present, there are almost no research papers on its activity. With the passage of time, literature in recent years has even considered 20α-DHP to be an inactive metabolite [16-18]. Due to the limited detection level in the 20th century, the accuracy of experimental data needs to be confirmed. Therefore, we re-evaluated the progestational activity of 20α-DHP and 20β-DHP, and the results showed that both 20α-DHP and 20β-DHP had progestational effects on endometrial stromal cells similar to P4 [19]. Therefore, it is necessary to further investigate the biological activities of 20α-DHP and 20β-DHP.

Since there are few research articles on 20β-DHP, this review is focused on 20α-DHP. In this paper, the published studies on the bioactivity of 20α-DHP are summarized, and the research progress in the synthetic pathway, content determination methods, bioactivity, and pharmacokinetic parameters of 20α-DHP is reviewed in detail so as to provide a literature basis for the study of 20α-DHP.

### 1.1 Search Strategy and Selection Criteria

We searched the relevant literature from 1950 to 2024 in PubMed and Web of Science databases with the keywords “dihydroprogesterone”, “20-alpha-dihydroprogesterone”, “20-α-hydroxyprogesterone”, “(20*R*)-20-hydroxypregn-4-en-3-one”, “20-beta-dihydroprogesterone”, “20-beta-hydroxyprogesterone”, “20beta-hydroxy-4-pregnen-3-one” and “progesterone metabolism”. We also used CAS numbers of 20α-DHP and 20β-DHP (145-14-2 and 145-15-3, respectively) to search for relevant information on the SciFinder database.

## 2 THE SOURCES OF 20α-DHP

### 2.1 Endogenous Sources

Steroid hormones are synthesized from cholesterol. During P4 synthesis, cholesterol enters the mitochondria *via* the steroidogenic acute regulatory protein and is converted to pregnenolone by the CYP11A1 enzyme. This steroid is then exported from the mitochondria and metabolized to P4 by the 3β-hydroxysteroid dehydrogenase (3β-HSD) in the endoplasmic reticulum [20].

The metabolizing enzyme of P4, 20α-HSD, is responsible for the conversion of P4 to 20α-DHP and belongs to one of the aldo-keto reductase (AKR) protein superfamily [21, 22]. The gene encoding this enzyme in humans is AKR1C1 (aldo-keto reductase family 1, member C1) [11], while in rats, it is AKR1C8 (aldo-keto reductase family 1, member C8) [23]. Another P4 metabolizing enzyme, 20β-HSD, reduces P4 to 20β-DHP [12, 13]. 20β-HSD is a member of the short-chain dehydrogenases/reductases (SDR) superfamily, also known as 3α, 20β-HSD, 3α/β, 20β-HSD, carbonyl reductase 1, and short-chain dehydrogenases [24, 25]. The *in vivo* synthetic pathway from cholesterol to 20α-DHP and 20β-DHP is shown in Fig. (**1**). In 1958, Zander *et al.* [8] found 20α-DHP and 20β-DHP in adipose tissue of menopausal women after P4 injection. 20β-HSD is present in many mammals [26, 27] and is involved in steroid metabolism, for *e.g.*, reducing 17α-hydroxyprogesterone to 17α,20β-dihydroxy-4-pregnen-3-one. There is no clear evidence in the literature to show that P4 is converted in humans by 20β-HSD to 20β-DHP, but both 20α-HSD and 20β-HSD are expressed in cattle, reducing luteinizing hormone (LH) to 20α-DHP and 20β-DHP [13, 28]. 20β-HSD is also frequently found in fish, where it is highly expressed at oocyte maturation [29, 30].

### 2.2 Exogenous Sources

#### 2.2.1 Chemical Synthesis

20α-DHP was synthesized by MacNevin *et al.* [31] using P4 as raw material, reduced by LiAlH_4_ to Pregn-4-ene-3,20-diol (3,20-DHP), and then selectively oxidized by MnO_2_ to 20-DHP. The synthesis route is shown in Scheme (**1**).

The yield of 20α-DHP obtained by this method was 57%. The yield ratio of 20α-DHP to 20β-DHP was 91:9 by thin layer chromatography-mass spectrometry, but the experiment did not separate the two diastereoisomers.

The two diastereoisomers were synthesized by Long *et al.* [32] using P4 as raw material through three steps of etherification, reduction, and de-etherization. The resultant route is shown in Scheme (**2**).

The yield ratio of 20α-DHP to 20β-DHP was about 0.35-0.40. The purity of 20α-DHP was 95%, whereas the purity of 20β-DHP was 99%.

#### 2.2.2 Biosynthesis

In addition to the chemical synthesis of 20α-DHP *in vitro*, biosynthesis is also involved. Naumann *et al.* [33] successfully converted P4 to 20α-DHP by functional expression of human AKR1C1 in fission yeast, which played a decisive role in large-scale *in vitro* synthesis of 20α-DHP. The conversion rate of 20α-DHP was 90 (±26) µM/d. The average yield of 20α-DHP was 300 µM/d and the total biotransformation time was 72 h in fed-batch fermentation under optimized reaction conditions. In this way, the toxicity and tedious experimental operation caused by chemical synthesis could be eliminated. This method avoided the complicated experimental operation and the possible environmental pollution caused by chemical reagents, providing a new way for the large-scale synthesis of 20α-DHP *in vitro*.

In summary, 20α-DHP can be obtained by chemical synthesis or biosynthesis. There is no comprehensive comparison between the two methods, and the difference between the yield and purity of the two is unclear.

## 3 CONTENT DETERMINATION

Since the discovery of 20α-DHP, researchers have been working on the determination of 20α-DHP in biological samples (blood, tissue fluids, tissue homogenates). In the 1950s and 1960s, the main determination methods used by researchers were paper chromatography-ultraviolet spectrophotometry (PC-UV) [34, 35] and paper chromatography-gas chromatography (PC-GC) [36].

In the 1970s, immunoassays were used for sensitive and specific steroid detection, with Abraham *et al.* being the first to use radioimmunoassay (RIA) for the detection of 17β-estradiol [37]. The RIA assay is inexpensive, rapid, and requires a small sample size for the assay, but it lacks unique specificity due to the potential for cross-reactivity of the antibody with other similar structural compounds [38-40]. In contrast, gas chromatography-mass spectrometry (GC-MS) and liquid chromatography-mass spectrometry (LC-MS) provide good linearity even at low concentrations, as well as superior selectivity for the separation and detection of steroids by chromatography-mass spectrometry [40]. Since steroids usually contain polar hydroxyl and ketone groups, GC-MS analysis requires the replacement of these functional groups with derivatization to obtain adequate volatility and thermal stability [41]. When using LC-MS, detection of analytes by electrospray ionization (ESI) or atmospheric pressure chemical ionization (APCI) eliminates this cumbersome preparation step [42]. Ultra-High-Performance Liquid Chromatography-High Resolution Mass Spectrometry UHPLC-HRMS allows for high sensitivity detection in the pg/mL range without derivatization, and the use of high-resolution targeted single ion monitoring mode allows for a selective analysis. Efficient solid-phase extraction methods and short chromatographic separation times enable high-throughput detection, so quantitative studies with large sample sizes can be easily performed [13].

The improvement of content determination methods largely depends on upgrading instruments and equipment. PC-UV and PC-GC have relatively low specificity and sensitivity, while the continuous emergence and upgrading of experimental tools, such as GC-MS, LC-MS, and UHPLC-HRMS have improved the specificity, accuracy, and sensitivity of measurement methods greatly. The qualitative leap of 20α-DHP content determination has also been realized. Studies on the difference between 20α-DHP and 20β-DHP *in vivo* have not been reported.

## 4 BIOLOGICAL ACTIVITY

### 4.1 Maintenance of Pregnancy

The ability of progestins to maintain pregnancy is usually evaluated by a fetal protection test. Wiest *et al.* [43] took ovariectomized mice as the test animals to investigate the effects of 20α-DHP or 20β-DHP on fetal survival in the womb through subcutaneous implantation and subcutaneous injection. The results suggested that 20α-DHP and 20β-DHP do not maintain pregnancy when administered subcutaneously or introduced as implants. Referencing the above experiments, Talwalker *et al.* [44] studied the effect of estradiol and progestins (P4, 20α-DHP, 20β-DHP) on the impact of fetal protection. The results demonstrated that neither 20α-DHP nor 20β-DHP could maintain the pregnancy of ovariectomized rats without mixing with estradiol. P4 at each dose had different levels of ability to maintain pregnancy. However, both showed a weak ability to maintain pregnancy when combined with estradiol, with 20α-DHP having about 1/15 of the ability of P4 and 20β-DHP having a stronger ability to maintain pregnancy than 20α-DHP.

Sawada *et al.* [45] investigated the relationship between P4 and 20α-DHP secretion in pregnant goats and their production sites in 1994. They demonstrated that the concentration of P4 and 20α-DHP in ovarian venous blood was significantly higher than that in the jugular vein at the end of gestation, indicating the ovary as the leading synthesis site of P4 at the end of pregnancy in goats. In addition, a sharp decrease in the concentration of P4 and a rapid increase in the concentration of 20α-DHP in ovarian venous blood were observed at the end of pregnancy, suggesting that 20α-DHP can terminate the pregnancy at the end.

Wiest *et al.* [46] explored in detail the mechanisms of regulation of physiological phenomena of the dramatic decline in P4 at the end of pregnancy. The team analyzed 20α-HSD’s activity in the ovaries of rats during pregnancy and measured the content of P4 and 20α-DHP in peripheral blood and uterine tissues. The experimental data showed that the activity of 20α-HSD and the concentration of 20α-DHP decreased gradually with the peak of P4 concentration in the early pregnancy of rats. The P4 level in peripheral blood increased a second time, accompanied by the gradual increase in 20α-DHP concentration in the second trimester (days 7 to 12). At this time, the activity of 20α-HSD did not increase significantly. P4 levels decreased sharply at the end of pregnancy, between days 19 and 21 (immediate delivery), the activity of 20α-HSD in the ovary increased gradually with the 20α-DHP concentration in peripheral blood, and a high positive correlation between 20α-DHP and 20α-HSD in plasma was observed. The corpora lutea gradually dissolved, and the activity of 20α-HSD increased logarithmically on day 21. Therefore, the decomposition of P4 to 20α-DHP is a normal regulatory mechanism by which the ovary reduces P4 levels during pregnancy in rats, and 20α-DHP plays a vital role in hormone regulation and the luteolysis process.

Similarly, in 2005, Piekorz *et al.* found that pregnancy termination was caused by 20α-HSD and the factor Stat5b related to 20α-HSD [47, 48]. Stat5 includes two signal transduction and transcriptional activators: Stat5a and Stat5b, which are major prolactin mediators signaling in the mammary gland and ovary, respectively. Mice lacking Stat5a had severely affected mammary gland development during their first pregnancy [49], and Stat5b deficiency was associated with early miscarriage [50]. A potential gene target of Stat5 transcriptional regulation is the P4 metabolizing enzyme 20α-HSD [51].

In summary, both 20α-DHP and 20β-DHP possess a weak ability to maintain pregnancy in the presence of estrogenic action. Although there is no direct evidence that 20α-DHP can terminate the pregnancy, the rapid increase in the concentration of 20α-DHP is indeed one of the signs of the end of pregnancy.

### 4.2 Endometrial Protection

In the 1950s, the endometrial protective effect of progestins was evaluated by endometrial proliferation assays (Hooker-Forbes test, Clauberg test). The rationale for this test was based on the fact that the endometrium showed a specific response to progestin in the form of hypertrophy of the interstitial nuclei of the ovary, and the degree of hypertrophy was used to evaluate the endometrial protective effect of progestin [52, 53]. Zander *et al.* [8] investigated the endometrial intensity of action of 20α-DHP and 20β-DHP using the Hooker–Forbes test. The mice showed specifically endometrial intensity of action after injection of 20α-DHP into a uterine horn, and the activity intensity was half that of the positive control P4 group. The 20β-DHP group was twice as active as P4. Clauberg’s experimental data showed that after 5 days of subcutaneous injection of 20α-DHP, estrogen-treated rabbits showed 1/3 intensity relative to the positive control P4 group, and the intensity of the 20β-DHP group was 1/5 to 1/10 times that of P4.

Forbes *et al.* [54] determined the minimum effective dose (MED) of several steroid hormones injected alone to achieve positive response in mice and explored and quantified the possible synergistic and antagonistic effects between P4 and other steroid hormones according to the principles of Hooker–Forbes and Clauberg tests. The results showed the MED of 20α-DHP to be smaller than that of P4 when administered alone, suggesting 20α-DHP’s activity to be higher than P4. In contrast, mixing 20α-DHP with P4 in any ratio did not show a significant endometrial response, and the strongest positive reaction was shown when P4 was mixed with 20β-DHP in a 4:1 ratio. This demonstrated that 20α-DHP may antagonize the endometrial response of P4 when administered in combination with P4, while the effect of 20β-DHP was the opposite of 20α-DHP.

Therefore, although the Hooker–Forbes and Clauberg tests are endometrial proliferation experiments, the results measured by the two methods differ when the endometrial intensity of action of 20α-DHP and 20β-DHP is measured, and the results determined by other people with the same experimental method may vary. This difference may be related to the animals or experimental conditions. However, both tests showed that 20α-DHP and 20β-DHP can promote endometrial hypertrophy and endometrial proliferation. 20α-DHP mixed with P4 can antagonize endometrial intensity of action. The endometrial intensity of action of 20β-DHP combined with P4 depends on the mixing ratio, and the appropriate mixing ratio can play a promoting role.

### 4.3 Regulation of Hormone Secretion and Activity

In the 1960s, some researchers suggested that mating stimulation can cause the rapid synthesis and release of 20α-DHP in the rabbit ovarian stromal tissue, and LH is the only pituitary hormone that causes this acute response [14, 55, 56]. The concentrations of P4 and 20α-DHP in the ovaries of female rabbits increased sharply after intravenous injection of LH [55]. Hilliard *et al.* [57] suggested that the amount of 20α-DHP could assess endogenous LH levels in the systemic circulation. Mated ovariectomized rabbits were divided into three groups. They were injected subcutaneously with estrogen, estrogen combined with 20α-DHP or 20α-DHP, and their ability to maintain LH release was tested. The results showed that mating stimulation increased the output of 20α-DHP and P4 slightly in the estrogen-induced ovariectomy female rabbits, namely, the temporary secretion of LH, but its content considerably decreased within a short time. Estrogen supplementation immediately after mating has been found to be ineffective in prolonging LH release. The study of Rasmussen *et al.* [58] showed the serum levels of LH to be maintained after injection of 20α-DHP oil solution alone, suggesting a relationship between the release of LH and 20α-DHP. Leyendecker *et al.* [59] believed that 20α-DHP cannot maintain the continuous secretion of LH when estrogen induction is absent, or estrogen is deficient. Therefore, a positive feedback regulation occurs between LH and 20α-DHP, and regulation can only occur during estrogen induction.

Subsequent studies have not supported the above results. In 1976, Goodman *et al.* [60] re-examined the function of 20α-DHP. They measured the changes of LH in the peripheral blood of animals pretreated with estrogen and ovariectomized (to eliminate the dramatic increase in ovarian P4) by radioimmunoassay. The data showed that mating stimulation caused a surge in LH in 10% of rabbits. Only 20% of animals pretreated with estrogen showed a surge in LH when injected with 20α-DHP immediately after ovariectomy. The result of 20α-DHP was not significantly different from conditions without 20α-DHP injection. These results suggested no necessary connection between LH secretion and 20α-DHP *in vivo*. Apparently, the findings of Goodman *et al.* were different from those of Hilliard *et al.* Goodman *et al.* demonstrated the relationship between LH and 20α-DHP more directly than Hilliard *et al.,* who evaluated the function of 20α-DHP through indirect data.

However, other studies have shown LH to inhibit the synthesis of 20α-DHP. Moon *et al.* [61] removed the pituitary gland from immature rats and pretreated them with oestradiol and follicle-stimulating hormone in 1986. Ovarian granulocytes were isolated, incubated with different doses of LH or human chorionic gonadotropin (HCG) for 24 h, and mixed with isotopically labeled P4. The effects of LH and HCG on P4 accumulation were observed. The results showed that P4 levels increased dose-dependently with the dosages of LH and HCG. However, the utilization rate of P4 and the content of 20α-DHP decreased dose-dependently with the dosage of LH and HCG, indicating that LH and HCG can up-regulate the content of P4 and inhibit the synthesis of 20α-DHP by inhibiting the metabolism of P4, which may be related to the inhibition of 20α-HSD.

Furthermore, there has been found a potential association between the action of 20α-DHP on the LH synthase 3β-HSD and LH secretion [45, 62-64]. Wiener *et al.* [65] measured the impact of 20α-DHP on 3β-HSD in placental tissues in 1967. The group of placental microsomes+20α-DHP+14C-labelled pregnenolone was the experimental group, and placental microsomes+14C-labelled pregnenolone co-incubation constituted the control group. It was found that pregnenolone could be converted into P4 in placental microsomes without 20α-DHP. Compared to the control group, the P4 production in the experimental group was significantly reduced, proving that 20α-DHP could inhibit P4 formation by down-regulating the content of 3β-HSD. Therefore, the aforementioned decrease in P4 levels at the end of pregnancy may also be caused by the down-regulation of 3β-HSD by 20α-DHP.

In conclusion, different researchers have different or even opposite conclusions about the regulatory effect of 20α-DHP on LH, which may be caused by other subjects and different times of experiment selection. 20α-DHP has also been reported to downregulate 3β-HSD, thereby inhibiting P4 synthesis.

It is reported that P4 can suppress lymphocyte proliferation by a mitogen [66-68]. Cell proliferation and differentiation involve multiple plasma membrane transport systems, among which is Na^+^/H^+^-exchange I (NHEI). This system causes cytosolic alkalinization and/or cell swelling in preparation for mitotic division [69, 70]. Conversely, cell shrinkage and cytosolic acidification are hallmarks of apoptosis. Chien *et al.* [68] found that P4 could stimulate cytosolic acidification in human T cells. To investigate whether this acidification was due to P4 inhibiting NHEI in T cells, the inactive steroid 20α-DHP was used as a control. The results demonstrated that P4 had an inhibitory effect on NHEI activity, while 20α-DHP had no such effect. Moreover, 20α-DHP could compete with P4 to relieve the inhibitory effect on NHEI. Therefore, 20α-DHP can regulate the inhibitory effect of P4 on T cells during pregnancy by inhibiting the activity of P4, and of course, the involvement of 20-HSD should not be ignored.

Ichikawa *et al.* [62] observed P4 compounds secreted by the ovaries of rats during the estrous cycle and pregnancy. The rat estrous cycle is divided into four stages: proestrus, estrus, metestrus, and diestrus. During the estrous cycle, 20α-DHP fluctuates significantly. Its concentrations are low in proestrus, reach a maximum in estrus, and then gradually decrease in metestrus and diestrus. The fluctuations in the concentration level of 20α-DHP are closely related to that of P4. From the end of proestrus to the end of estrus, the P4 level decreases rapidly, while the level of 20α-DHP gradually increases, as a result of the action of 20α-HSD [71]. During the rat estrous cycle, 20α-HSD expression was extremely low in the newly formed corpus luteum from estrus to diestrus, followed by a gradual increase. This expression pattern coincided with the timing of luteal function degeneration, suggesting 20α-HSD to play a triggering role in luteal function degeneration [72]. In addition, the expression of 20α-HSD was regulated by prostaglandin F2α (PGF2), a key factor in luteal degeneration, triggering luteal degeneration by stimulating the expression and enzymatic activity of the 20α-HSD gene, leading to an increase in the metabolism of P4, and consequently to a decrease in P4 levels in the corpus luteum. Correspondingly, the human menstrual cycle is divided into four phases: the follicular phase, the ovulatory phase, the luteal phase, and the menstrual phase. During the menstrual cycle, significant changes in 20α-DHP mainly occur after the LH surge, during the follicular phase, especially on the day and two days after the LH surge, when its level increases significantly and highly correlates with changes in P4 levels [73]. From the beginning of rat pregnancy to day 10, the concentration of 20α-DHP gradually decreased to the lowest level, slowly increased from day 10 to day 22, and rapidly increased to the maximum from day 20 to day 22 (the end of pregnancy). Meanwhile, the concentration of P4 decreased from a high level on day 15 to a low on day 22. In humans, from the beginning of pregnancy to week 9, the concentration of P4 slightly increased first and then decreased, and then showed an increasing trend until week 39 and began to decline. The concentration of 20α-DHP fluctuated steadily from the beginning of pregnancy to week 20, and then generally increased from week 21 until the end of pregnancy, without a decreasing trend [74]. This could be a result of the activation of 20α-HSD by PGF2α during pregnancy, metabolizing P4 to 20α-DHP, regulating the concentration of P4, and reducing P4 toxicity in cells to maintain normal pregnancy [75-77].

### 4.4 Ovulation-Promoting Effect

Throughout the rat estrus cycle, 20α-DHP is the dominant P4 in content [78-81]. During estrus, follicles are formed. To investigate whether 20α-DHP has an ovulatory effect, Gilles *et al.* [82] investigated the effects of the subcutaneous injection of different concentrations of 20α-DHP on ovulation in female juvenile rats pretreated with non-ovulatory doses of pregnant mares serum gonadotropin. The results showed that low doses of 20α-DHP did not induce ovulation, whereas high doses of 20α-DHP showed a level of ovulation. In conclusion, 20α-DHP can induce ovulation. Although the ovulation effect is weak, this activity is still essential.

### 4.5 Regulation of Uterine Epithelial Ciliary Hyperplasia

Conti *et al.* [83] explored the effects of subcutaneous injection of 20α-DHP on the proliferation and differentiation of the rabbit endometrial membrane in the context of studies on the regulation of ovarian hormone in the proliferation and differentiation of the rabbit endometrial membrane [84-86]. Conti *et al.* found that 20α-DHP induced the formation of large white cells in the endometrial epithelium of ovariectomized rabbits, with about 190 white clear cells in 1000 cells, compared to about 1‰ white cells in the P4 group [87]. Electron microscopy showed that the white cells had cilia but no mitochondria, endoplasmic reticulum, or ribosomes [83]. Given that the ovaries of the tested animals were removed, the effect of other endogenous hormones was eliminated, so the results suggested that 20α-DHP alone can produce a ciliating effect. In addition, this experiment showed that this effect was antagonized by 20α-DHP in combination with P4, so no ciliated cell formation occurred. Since the mechanism of formation and the physiological role of these ciliated cells are unknown, their experimental morphological and cytochemical evidence suggests that the hyaline cells are late-differentiated ciliated cells with signs of death. Moreover, no other necrotic cells were observed in the investigation, so the authors suspected the transformation mechanism of rabbit endometrial cells to mainly be shedding. Schueller *et al.* [88] found that although 20α-DHP leads to ciliated cell formation during early pregnancy and oestrus, these ciliated cells gradually disappear under P4 antagonism, suggesting endometrial regulation to also be associated with 20α-DHP.

### 4.6 Antagonism of Breast Cancer

Estrogen stimulates the proliferation of breast cancer cells by inducing 5α-reduced metabolites [89]. Pasqualini *et al.* [90] investigated the anti-aromatase effect of P4 and its metabolites 5α-dihydroprogesterone (5α-DHP) and 20α-DHP in MCF-7aro breast cancer cell lines. MCF-7aro breast cancer cell lines showed high activity of aromatase, a rate-limiting enzyme for oestradiol synthesis that converts testosterone into oestradiol. MCF-7aro breast cancer cells were incubated separately with physiological concentrations of 3H-labelled testosterone as a control. P4, 20α-DHP, or 5α-DHP were added as three experimental groups based on the control group. Their effects on testosterone conversion were evaluated by measuring each group’s ratio of testosterone to oestradiol content. The results demonstrated that the percentage of testosterone to oestradiol in the 20α-DHP group was the highest (1.481), followed by that of the P4 group (0.705) and that of the 5α-DHP group (0.496) at the same dose concentration (5×10^-8^ mol/L) compared to the control group, whereas the ratio in the control group was only 0.430. The percentage of testosterone to oestradiol was greater in the 20α-DHP group (2.582), virtually unchanged in the P4 group (0.707), and decreased in the 5α-DHP group (0.368) when the dose of the three hormones was increased to 5×10^-6^ mol/L. This result indicated that 20α-DHP could prevent breast cancer by being an anti-aromatase agent with an anti-proliferative effect on breast cancer cells.

### 4.7 Central Nervous System Effect

Nowak *et al.* [15] investigated the uptake and transformation of ^3^H-labeled 20α-DHP in the central nervous system and peripheral tissues after tail vein injection in rats, and the compounds were separated from tissues or plasma by scintillation counting localization and thin-layer chromatography, and the content of the separated substances was determined by GC or LC. Experimental data showed that 20α-DHP was distributed in several regions after injection, including the pineal gland, preoptic area of the hypothalamus, medial basal hypothalamus, midbrain, cerebellum, cerebral cortex, anterior pituitary, peripheral blood, adrenal gland, liver, uterus, and skeletal muscle. However, its central physiological role is unclear and must be further explored. The ability of central nervous system regions to selectively absorb and retain 20α-DHP suggests that 20α-DHP may have central nervous system effects, while the specific central effects remain to be further determined.

### 4.8 Diagnostic Indicators of Psoriasis and Polycystic Ovary Syndrome

As there are no clear criteria for the diagnosis of psoriasis, currently the diagnosis is made with the help of specific biomarkers. Kishikawa *et al.* [91] used metabolomics analysis in order to identify potential biomarkers for psoriasis and its subtypes. They constructed a large number of plasma samples for metabolomics analysis by an untargeted metabolomics approach, and drew conclusions from the psoriasis-control correlation test; 20α-DHP was significantly lower in psoriasis samples than in controls, in addition to which three metabolites significantly different from controls were detected. These differential metabolites may be used as potential biomarkers for psoriasis and its clinical subtypes, contributing to the understanding of the pathophysiology of psoriasis.

Polycystic ovary syndrome (PCOS) is defined as hyperandrogenism and ovarian dysfunction and lacks a specific physiological diagnosis. Altinkilic *et al.* [16] performed a comprehensive serum steroid analysis in women with PCOS using LC-MS and the experimental results showed that patients with PCOS had the lowest levels of 20α-DHP as compared to healthy controls. This provides a basis for the diagnosis of PCOS and also lays the foundation for recognizing PCOS.

## 5 PHARMACOKINETIC CHARACTERISTICS

### 5.1 Distribution and Metabolism

In 1974, Nowak *et al.* [92] incubated isotopically labeled 20α-DHP with rat medial hypothalamic basal tissues *in vitro*, and qualitative and quantitative analyses of the incubated tissue homogenates by GC revealed the radioactive compounds to be (20*S*)-20- -5α-pregnan-3-one (21%), (3*S,* 20*S*)-5α-pregnane-3, 20-diol (3%), and 20α-DHP (69%). 20α-DHP could be reduced to 20α-hydroxy-5α-pregnen-3-one by 5α-reductase and to 5α-pregnene-3α,20α-diol by 3α-hydroxysteroid dehydrogenase [13], and the specific metabolic pathways are shown in Fig. (**2**). *In vitro* experiments by Nowak *et al.* [93] demonstrated the metabolites of 20α-DHP in the anterior pituitary of rats to be consistent with the metabolites in the medial basal hypothalamus.

### 5.2 Pharmacokinetic Parameters

Currently, the pharmacokinetic parameters of 20α-DHP were derived from indirect studies. Zhang *et al.* [94] determined the content of 20α-DHP in the plasma of rats after intramuscular injection of P4 by High-Performance Liquid Chromatography, and calculated the pharmacokinetic parameters. The following results were obtained: 1) the half-life was 3.3±1.9 h and the maximum concentration was 218±41 μg/l; 2) the peak time was 3.2±0.4 h; and 3) the atrioventricular model was combined with a two-compartment model. The peak time of 20α-DHP was similar to that of P4 compared to the blood concentration-time curve of P4, indicating P4 to be quickly converted to 20α-DHP after absorption. The amount of 20α-DHP transferred into the uterus in plasma was less than that of P4. The concentration of 20α-DHP was significantly higher than that of P4 at the beginning of oestrus in female rats [62]. The surge effect may be related to the rapid metabolism of P4 and the secretion of 20α-DHP. The early surge effect suggested 20α-DHP to be involved in some physiological functions.

## CONCLUSION

This review has described the synthetic pathway, content determination method, biological activity, and pharmacokinetic parameters of 20α-DHP.

20α-DHP is metabolized *in vivo* from P4 by 20α-HSD, an endogenous metabolite of P4 that can be chemically and biosynthesized *in vitro*. Although chemical synthesis routes have been available for splitting 20α-DHP and 20β-DHP, the limitations of chemical synthesis do not allow for a one-step conversion to one conformation. This chemical synthesis is relatively complex, whereas the biosynthesis method is more selective and capable of directly producing compounds in specific conformations. Although there is no data to show that this method has a higher yield of 20α-DHP with higher purity than chemical synthesis, the method is green and has mild reaction conditions, and is bound to have a wider application.

The content determination methods of 20α-DHP were mainly based on PC-UV and PC-GC in the 1950s and 1970s, and the RIA method has been found to have higher specificity since the 1970s. With the development of the times, GC-MS, LC-MS and UHPLC-HRMS have been gradually used, and the methods for content determination have been gradually improved.

From the 1960s to the 1990s, a lot of research has been carried out on the biological activities of 20α-DHP, which include maintaining pregnancy, protecting the endometrium, regulating the secretion of hormones, regulating the proliferation of uterine epithelial cells, *etc*. Due to the limitations of the times, some of the early assays have been outdated and the reliability of the data has been questionable; thus, these bioactivities need to be reevaluated. In the 21^st^ century, scholars’ research on the bioactivity of 20α-DHP has mainly focused on antagonizing breast cancer, central effects, and as a test indicator for psoriasis and polycystic ovary syndrome.

Although the pharmacokinetic parameters of 20α-DHP have been obtained through indirect experiments, the data on maximum blood concentration and half-life, which help to determine the appropriate physiologic dose of 20α-DHP and the time interval of administration, provide a reference for conducting direct experiments.

In conclusion, there are still many unclear aspects in the study of 20α-DHP, and the biological activity and the process of absorption, distribution, metabolism, and excretion *in vivo* need to be further explored, and whether further research has value needs to be scientifically evaluated by comprehensive and systematic experiments.

## Figures and Tables

**Fig. (1):**
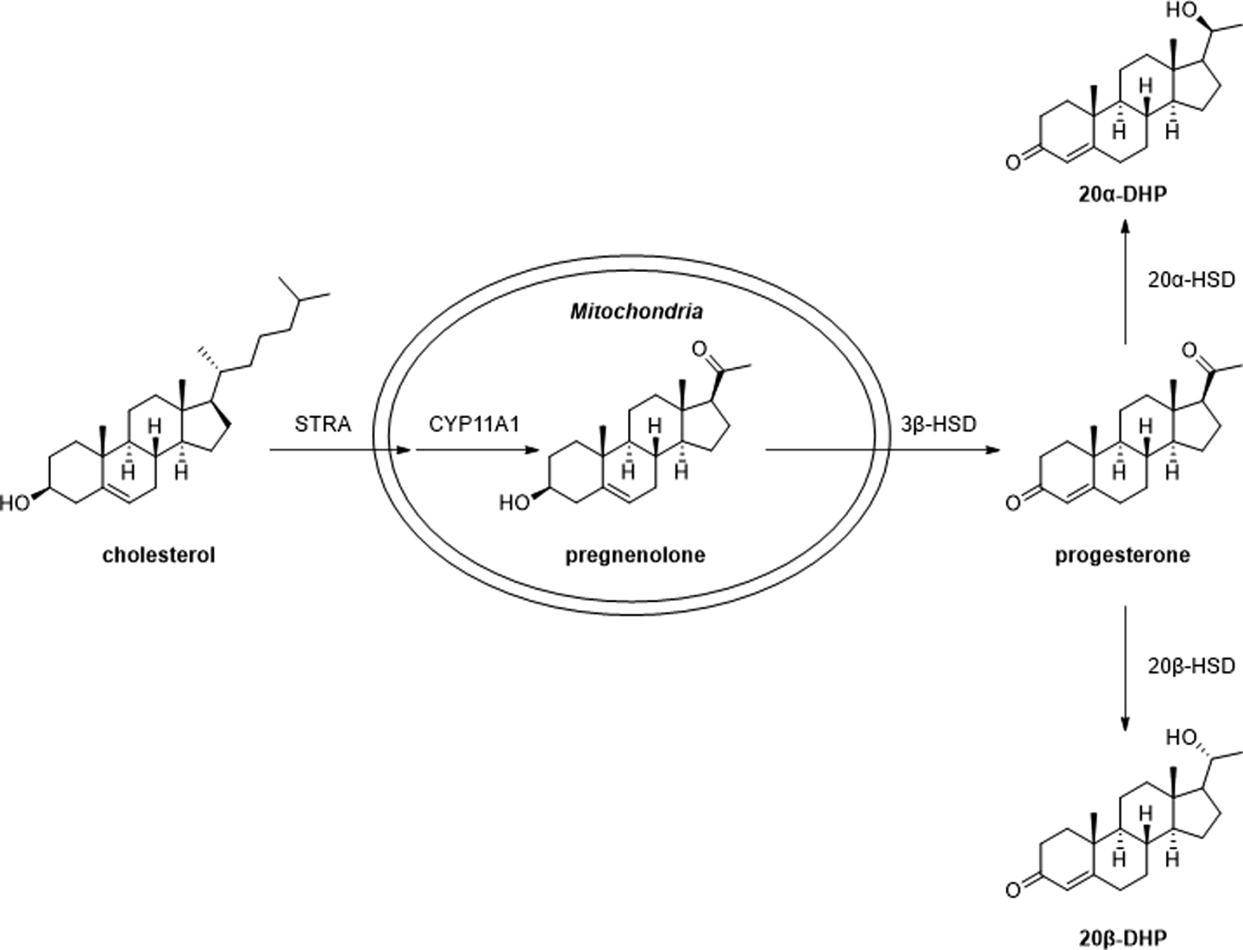
The *in vivo* synthetic pathway from cholesterol to 20α-DHP and 20β-DHP.

**Scheme 1:**
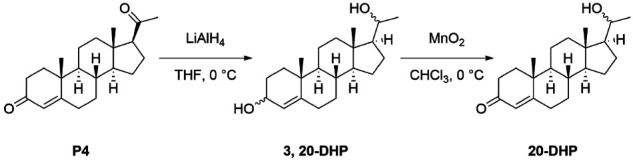
Synthesis route of 20-DHP (MacNevin *et al.’s* method).

**Scheme 2:**
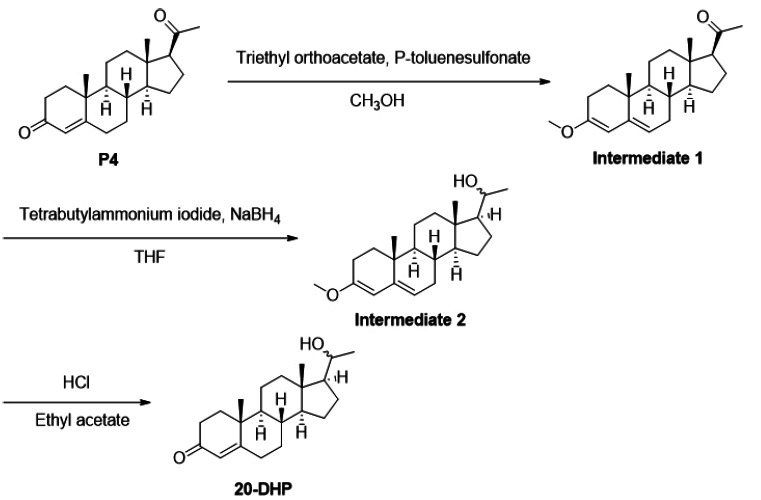
Synthesis route of 20α-DHP and 20β-DHP (Long *et al.’s* method). (Intermediate 1: 1-[(8S,9S,10R,13S,14S,17S)-3-methoxy-10,13-dimethyl-2,7,8,9,10,11,12,13,14,15,16,17-dodecahydro-1H-cyclopenta[a]phenanthren-17-yl] ethan-1-one. Intermediate 2: 1-[(8S,9S,10R,13S,14S,17S)-3-methoxy-10,13-dimethyl-2,7,8,9,10,11,12,13,14,15,16,17-dodecahydro-1H-cyclopenta[a]phenanthren-17-yl] ethan-1-ol).

**Fig. (2):**
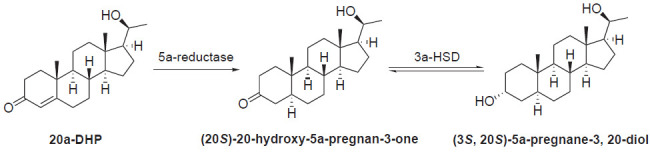
The metabolic pathway of 20α-DHP.

## LIST OF ABBREVIATIONS

P4: Progesterone
20β-DHP: 20β-Hydroxyprogesterone
20α-HSD: 20α-Hydroxysteroid Dehydrogenase
20β-HSD: 20β-Hydroxysteroid Dehydrogenase
AKR: Aldo-Keto Reductase
AKR1C1: Aldo-Keto Reductase Family 1, Member C1
AKR1C8: aldo-Keto Reductase Family 1, Member C8
3,20-DHP: Pregn-43,2-ene-0-diol
PC-UV: Paper Chromatography-Ultraviolet Spectrophotometry
PC-GC: Paper Chromatography-Gas Chromatography
GC-MS: Gas Chromatography-Mass Spectrometry
RIA: Radioimmunoassay
LC-MS: Liquid Chromatography-Mass Spectrometry
UHPLC-HRMS: Ultra-High-Performance Liquid Chromatography-High Resolution Mass Spectrometry
MED: Minimum Effective Dose
HCG: Human Chorionic Gonadotropin
NHEI: Na^+^/H^+^-exchange
PGF2: Prostaglandin F2α
5α-DHP: 5α-dihydroprogesterone
PCOS: Polycystic Ovary Syndrome
LH: Luteinizing Hormone
SDR: Short-Chain Dehydrogenase/Reductase
3β-HSD: 3β-Hydroxysteroid Dehydrogenase
20β-HSD: 20β-Hydroxysteroid Dehydrogenase
20α-DHP: 20α-Hydroxyprogesterone
